# On the Characterization and Correlation of Compositional, Antioxidant and Colour Profile of Common and Balsamic Vinegars

**DOI:** 10.3390/antiox7100139

**Published:** 2018-10-11

**Authors:** Vassilia J. Sinanoglou, Panagiotis Zoumpoulakis, Charalambos Fotakis, Nick Kalogeropoulos, Aikaterini Sakellari, Sotirios Karavoltsos, Irini F. Strati

**Affiliations:** 1 Laboratory of Chemistry, Analysis & Design of Food Processes, Department of Food Science and Technology, University of West Attica, Ag. Spyridonos, 12243 Egaleo, Greece; v_sinanoglou@yahoo.gr; 2 Institute of Biology, Medicinal Chemistry & Biotechnology, National Hellenic Research Foundation, 48, Vas. Constantinou Ave., 11635 Athens, Greece; pzoump@eie.gr (P.Z.); bfotakis@yahoo.com (C.F.); 3 Department of Dietetics-Nutrition, School of Health Science and Education, Harokopio University of Athens, Eleftheriou Venizelou 70, 17676 Kallithea, Greece; nickal@hua.gr; 4 Laboratory of Environmental Chemistry, Department of Chemistry, National and Kapodistrian University of Athens, Panepistimioupolis, Zografou, 15784 Athens, Greece; esakel@chem.uoa.gr (A.S.); skarav@chem.uoa.gr (S.K.)

**Keywords:** vinegars, phenolics’ profile, antiradical and antioxidant activity, colour, GC-MS, FT-IR and NMR spectroscopy, principal component analysis

## Abstract

Commercially available common and balsamic vinegars were examined, using a combination of spectrophotometric, chromatographic, colorimetric and spectroscopic methods. Total phenolic content, antioxidant activity, radical scavenging capacity, phenolic profile, colour parameters, Fourier Transform Infrared (FT-IR) absorbance spectra and Nuclear Magnetic Resonance (^1^H NMR) spectra were comparatively studied. The main scope was the assessment of vinegar antioxidant and metabolic profiles and the identification of the most appropriate features influencing their type and subtypes. Red grape balsamic vinegars exhibited the strongest antioxidant profile. High total phenolic content and radical scavenging-antioxidant activity of vinegars was strongly correlated with high hue-angle and colour density values and low lightness and a* values. FT-IR spectra analysis confirmed the presence of organic acids and carbohydrates and, in combination with Gas Chromatography-Mass Spectrometry (GC-MS), the occurrence of phenolic compounds. NMR spectroscopy enabled the identification of 27 characteristic metabolites in each type of vinegar. The combination of all applied techniques provides critical information on compositional differences among the vinegars and could serve as an application tool for similar fermentation products.

## 1. Introduction

Vinegar is a widespread ingredient of the diet and used as acidic seasoning in salads, sauces and cooking, to improve the taste and the flavour of foods. In Ancient Greece vinegars were used as medicine and antiseptic agents. Vinegar is a fermentation product which can be produced by conventional and rapid methods from various fruits and vegetables, as grape, apple, pomegranate, sugarcane and rice [1]. Vinegars are commercially grouped as wine (red or white) and fruit vinegars. Based on their production from wine or cooked grape must fermentation, they are classified in common and balsamic, respectively. 

Recent studies have shown that the particular composition of vinegar, especially regarding organic acids, polyphenols and other phenolic compounds, affect the final characteristics and, hence, the quality of a vinegar and may have a positive effect for human health due to their antioxidant activity [1,2]. Therefore, the consumption of vinegar may play a role in the prevention of several diseases like hypertension, cardiovascular illnesses, cancer, diabetes and others [3,4,5]. 

A wide range of techniques have been applied for the analysis and discrimination of vinegars; among them spectroscopic, colorimetric and chromatometric have demonstrated to be an excellent alternative to other more time and reagent-consuming methods. In this sense, UV-Vis and infrared absorption spectroscopy techniques can be employed to assess the antioxidant and antimicrobial potential of vinegars as well as to identify bioactive compounds (organic acids, polyphenols, flavonoids and others) that discriminate vinegars from diverse origins [6,7,8,9]. Moreover, ^1^Η-NMR has been utilized for the differentiation of various types of cereal and grape vinegars in relation to their metabolic profiles [10,11,12], as well as GC-MS and GC-FID were used to assess the volatile profile of vinegars and determine metabolites evident of natural vinegars [13,14].

As only few studies have been carried out on vinegars regarding their quality evaluation and valorisation, the aim of this study was to assess the compositional, antioxidant and colour profile of common and balsamic vinegars available in the market, using a combination of spectrophotometric, chromatographic, colorimetric and spectroscopic methods. The results of the study could provide useful information regarding functional ingredients, physicochemical and bioactive properties as well as nutrient assessment of common and balsamic vinegars. 

## 2. Materials and Methods 

### 2.1. Chemicals, Standards and Solvents

All reagents and solvents used were of analytical grade and they were purchased from Mallinckrodt Chemical Works (St. Louis, MO, USA), Alfa Aesar GmbH & Co (Karlsruhe, Germany) and Sigma-Aldrich Chemie GmbH (Taufkirchen, Germany). NMR solvents and standards D_2_O (99.9%), NaN3 (99.5%) and TSP-d4 (97%) were purchased from Sigma Chemical Co. (St. Louis, MO, USA). Folin–Ciocalteu’s phenol reagent and Trolox (6-hydroxy-2,5,7,8-tetramethylchroman-2-carboxylic acid) were obtained from Sigma Chemical Co. (St. Louis, MO, USA), ABTS [2,20-azinobis(3-ethylbenzothiazoline-6-sulfonic acid)] from Tokyo Chemical Industry Co. Ltd (Tokyo, Japan) and 3,4,5-trihydroxybenzoic acid from Alfa Aesar (Karlsruh, Germany). 

### 2.2. Sampling

Twenty-three common and twenty balsamic vinegars from different producers were purchased. Vinegars were further grouped according to their colour (red or white) and origin (grape or other fruit). The information provided on the bottle label is listed in Table 1.

### 2.3. Determination of Total Phenolic Content (TPC)

The total phenolic content of each sample was determined applying a modified micro method of Folin–Ciocalteu’s colorimetric assay according to Andreou et al. [15]. The absorbance was measured at 750 nm with a Vis spectrophotometer (Spectro 23, Digital Spectrophotometer, Labomed, Inc., Los Angeles, CA, USA). The total phenolic content was expressed as mg gallic acid equivalents (GAE) per L of vinegar.

### 2.4. Scavenging Activity on 2,2′-azino-bis-(3-Ethylbenzothiazoline-6-Sulfonic Acid) Radical (ABTS^●+^)

The radical scavenging activity of vinegar samples was determined according to the method described by Lantzouraki et al. [16]. Absorbance was measured at 734 nm with a Vis spectrophotometer (Spectro 23, Digital Spectrophotometer, Labomed, Inc., Los Angeles, CA, USA). The radical scavenging activity of the samples was expressed as mg Trolox equivalents (TE) per L of vinegar.

### 2.5. Ferric Reducing/Antioxidant Power Assay (FRAP) 

The ferric reducing antioxidant power assay, based on the reduction of a ferric-2,4,6-tripyridyl-s-triazine complex to the ferrous form, was carried out according to the method described by Lantzouraki et al. [17]. The absorbance was measured at 595 nm, on a Vis spectrophotometer (Spectro 23, Digital Spectrophotometer, Labomed, Inc., Los Angeles, CA, USA). Results were expressed as mg FeSO_4_·7H_2_O per L of vinegar.

### 2.6. Determination of Individual Phenolic Compounds by GC-MS

Simple phenolic compounds were isolated from vinegar samples by solid phase extraction (SPE) on C8 Isolute columns, essentially as described by Soleas et al. [18]. Columns were preconditioned with ethyl acetate (3 mL), methanol (3 mL) and ultrapure water (3 + 2 mL). Then, vinegar samples (100 μL) were loaded on the SPE columns and allowed to drain by gravity. Solvent was subsequently removed under reduced pressure and the phenolics were recovered by ethyl acetate (3 mL). After solvent removal in a centrifuge evaporator (Speed Vac) the residues were dissolved in methanol (500 μL) and aliquots of the methanolic solutions were evaporated to dryness and silylated with *N*,*O*–bis-trimethylsilyl-trifluoroacetamide (BSTFA). Simple phenolics in the form of trimethylsilyl (TMS) ethers, were quantitated by selective ion monitoring GC-MS employing 3-(4-hydroxyphenyl)-1-propanol as internal standard [19]. The target and qualifier ions for the trimethylsilyl ethers (TMS) of the phenolic compounds and the internal standard (IS) are given in Table S1. 

### 2.7. Colour Measurement

Colour values such as L* (lightness), a* (redness/greenness), b* (yellowness/blueness) and h (hue angle in degrees) were measured with a tristimulus chromatometer (model CR-400, Minolta, Tokyo, Japan) calibrated with a white standard plate (L*: 97.83, a*: −0.45, b*: +1.88). Three random readings per sample were taken and averaged.

The colour characteristics of vinegars were determined by measuring the absorbance spectrophotometrically at 420 nm (yellow) and 520 nm (red), using a 1 cm path length cuvette. The absorbance was measured on a double-beam UV–vis spectrophotometer Hitachi U-3210 (Hitachi Ltd., Tokyo, Japan). Colour density and tint were calculated using the following equations:

Colour Density = A420 + A520

Colour Tint = A420/A520

### 2.8. FT IR Spectroscopy

FT-IR spectra were collected with an Alpha- P spectrometer, the Alpha FT-IR wine analyser (Bruker Optics Inc.) (Billerica, MA USA) on a diamond ATR crystal covered with a flow through cell, facilitating sample injection. The Alpha-P instrument has a potassium bromide (KBr) beam splitter and a 2 × 2 mm temperature controllable ATR diamond crystal sample plate, which was set at 40 °C. The instrument was fitted with OPUS software (OPUS version 7.2 for Microsoft Windows, Bruker Optics). No further sample preparation was done for spectral analysis and volumes of 5 mL were used. The spectrum of each sample and background were obtained from 4000 to 375 cm^−1^ and the average of 64 scans at a resolution of 8 cm^−1^ with a scanner velocity of 7.5 kHz was recorded. One background measurement was taken before each sample measurement. The ALPHA Wine Analyser comes with a starter calibration that was assembled by the accredited (DAkkS) Institute Heidger (Kesten, Germany). The organic acid and sugar contents were measured for each vinegar sample using the “ALPHA wine analyser” apparatus. 

### 2.9. NMR Spectroscopy

#### 2.9.1. Sample Preparation for NMR Analysis

300 μL of each vinegar sample were diluted with 150 μL D_2_O, then mixed with 50 μL of buffer (pH 5.6 in D_2_O containing 0.1% of TSP and 0.013% of sodium azide) and transferred into a 5 mm-NMR sample tube.

#### 2.9.2. NMR Measurements

^1^H NMR spectra were acquired with the 1D NOESYPRESAT pulse sequence. The receiver gain was set at 30; two hundred and fifty-six transients were collected with 72 K data points using a spectral width of 9615.4 Hz with a relaxation delay of 2 s, mixing time of 100 ms and an acquisition time of 4.00 s.

A series of 2D experiments, gCOSY, zTOCSY, gHMBCad, gHSQCad were recorded at 25 °C and permitted the assignment of the existing metabolites. The interpretation of 2D spectra was performed with the use of MestReNova v.10.1 software. The identification procedure was based on 2D NMR spectra, the Chenomx Suite 7.0 reference ^1^H NMR metabolic database and literature data [12,13]. 

#### 2.9.3. NMR Data Reduction and Spectral Alignment

The NMR spectral data was reduced into spectral buckets of 0.0001 ppm. The following regions were removed: D_2_O (4.6–4.8 ppm), acetic peak (1.9–2.1 ppm). The spectra were then normalized to the standardized area of the reference compound and converted to ASCII format. The ASCII format files were imported into MATLAB (R2006a, Mathworks, Inc. 2006, Natick, MA, USA) and all spectra were aligned using the Correlation Optimized Warping (COW) method [20].

### 2.10. Statistical Analysis

#### 2.10.1. Univariate Data Analysis

All the IR spectroscopic, the spectrophotometric assays and colorimetric measurements were carried out in triplicate for all samples. The colour values were treated as dependent variables and analysed using one way analysis of variance. The values for total phenolic content, radical scavenging and antioxidant activity did not meet the ANOVA assumptions (i.e., normal distribution and homogeneity of variances within groups), thus the Kruskal–Wallis non-parametric test was performed. The normality was tested by performing the Kolmogorov–Smirnov test. Correlations were performed using the Spearman’s rank-order correlation coefficient, which is a nonparametric measure of the strength and direction of association that exists between variables. In addition, one-way ANOVA was used to compare statistically test the NMR data in terms of the vinegar type. All statistical calculations were performed with the SPSS package (IBM SPSS Statistics, version 19.0, Chicago, IL, USA) for Windows. 

#### 2.10.2. Multivariate Data Analysis

The IR data set were imported into the SIMCA-P version 14.0 (Umetrics, Umeå, Sweden) for statistical analysis. The exploratory principal component analysis (PCA) was applied to acquire a general insight and visualize any relation (trends, outliers) among the observations (samples) [21]. Loading and contribution plots were extracted to reveal the variables that bear class discriminating power. All models were Pareto (Par) scaled and extracted at a confidence level of 95%. The quality of the models was described by the goodness-of-fit R^2^ (0 ≤ R^2^ ≤ 1) and the predictive ability Q^2^ (0 ≤ Q^2^ ≤1) values [22].

## 3. Results and Discussion

### 3.1. Properties of Bioactive Compounds of the Vinegars

The data concerning the total phenolic content (TPC), radical scavenging and antioxidant activity of the forty-three studied vinegars as well as their descriptive statistics are reported in Table 2, Tables S2 and S3.

The great fluctuation of the results and the high values of standard deviations indicate a significant variability of the phenolic profile of the studied vinegars. According to the literature [23,24], several factors affect the vinegar quality and composition such as the raw material used (grape, apple, etc.), the fruit composition as a function of the cultivar and agricultural conditions, the vinegar processing technology including the cooking process, the fermentation and the acetification process and the aging period. Hence, red grape balsamic vinegars (RGBV) demonstrated significantly higher TPC (*p* < 0.05), radical scavenging and antioxidant activity compared to the white grape balsamic (WGBV), red grape (RGV) white grape (WGV) and fruit (FV) vinegars (Table 2). Even though RGBV exhibited higher TPC (*p* < 0.05) than red grape balsamic vinegars with honey (RGBVH), no significant variation in radical scavenging and antioxidant activity was observed. According to Almaraz-Abarca et al. [25], the radical scavenging and antioxidant activity of honey is more dependent on the flavonoid profile than on the total flavonoid content. Hence, the specific phenolic constituents of honey may have contributed to the radical scavenging and antioxidant activity of the vinegars and not the total phenolic content. Furthermore, the red grape vinegars (RGV) presented higher (*p* < 0.05) TPC, scavenging and antioxidant activity than the white grape (WGV) and fruit (FV) vinegars. This result could be correlated with the total phenolic content of the raw materials (red or white grapes and fruits, respectively) since their phenolic composition contributes to the phenolic profile of vinegar. Additionally, RGV exhibited higher (*p* < 0.05) TPC and scavenging activity than WGBV, whereas no significant variation in antioxidant activity was observed (Table 2). Interestingly, sea buckthorn vinegar (F5) showed the highest (*p* < 0.05) TPC (428.37 ± 10.79 mg gallic acid E/L), scavenging activity (496.49 ± 13.84 mg Trolox E/L) and ferric reducing power (2409.87 ± 154.55 mg FeSO_4_·7H_2_O/L), compared to all white wine, apple and pomegranate common vinegars (Table S3). Furthermore, although sea buckthorn vinegar exhibited higher TPC (*p* < 0.05) than red wine vinegars, it did not exhibit a similar result regarding the radical scavenging and antioxidant activities (Table S3). In conjunction with the above findings, Negi and Dey [26] reported that sea buckthorn wine presented higher or similar total phenolic content compared to red grape wine and higher compared to apple wine. Moreover, Eccleston et al. [27] reported that sea buckthorn juice was found to be a rich source of vitamin C, vitamin E, carotenoids and flavonoids. The Modena balsamic red from Altis (Unilever) (BR6) showed the highest (*p* < 0.05) TPC (2867.33 ± 61.10 mg gallic acid E/L), radical scavenging (4417.44 ± 52.22 mg Trolox E/L) and antioxidant activity (26293.23 ± 128.99 mg FeSO_4_·7H_2_O/L), compared to all samples, followed by the ageing balsamic vinegar Aceto Botanico Adriani Gold Seal from Lazaridi (BR10) (Table S2). The wine white vinegar from Meteora (Agricultural Cooperative of Trikala) (sample WW3) revealed the lowest (*p* < 0.05) radical scavenging and antioxidant activity, whereas the pomegranate vinegar from Ayanoglou S.A. (sample F4) exhibited the lowest (*p* < 0.05) TPC, compared to all samples. The apple vinegars (samples F1–F3) (Table S3) revealed low contents for total phenolic compounds compared to most of the common vinegars, which could be attributed to the low phenolic content of the raw material used, in accordance with Sakanaka and Ishihara [28] findings. 

Generally, the considerably higher fluctuations of antioxidant activity values observed when compared to the respective TPC and radical scavenging activity values, could be possibly attributed to the different phenolic compounds’ profile of the samples, which is in accordance to Dávalos et al. [29] and to Ozturk et al. [30] findings. 

Nevertheless, high positive Spearman correlation values were observed between TPC and radical scavenging activity, TPC and antioxidant activity as well as among radical scavenging and antioxidant activity (Table 3), confirming the contribution of phenolic constituents (irrespectively to their profile) to the radical scavenging and antioxidant activity of the vinegars.

### 3.2. Phenolic Compounds’ Profile of the Vinegars

The main phenolic compounds determined in the studied vinegar samples were phenolic acids and flavonoids (Tables S4 and S5). In particular, a total of 21 compounds were determined in the balsamic and 23 in the common vinegars. Gallic acid predominated in most samples followed by caffeic acid. Protocatechuic, cinnamic, vanillic, ferulic, *p*-hydroxybenzoic, *p*-hydroxyphenylacetic and syringic acids were detected in almost all balsamic and common vinegars. According to Gil-Muñoz [31] the extraction of the aforementioned acids is depended on the ethanol content during the alcoholic fermentation. The most interesting findings resulting from the comparative study of the vinegars’ phenolic profile, were provided as proportions of the total quantified phenolics (Tables S4 and S5) and are discussed below. Tyrosol proportion was higher in all common and white balsamic vinegars, compared to most of red balsamic ones. Tyrosol derives from the amino acid (tyrosine) metabolism of yeast cells during alcoholic fermentation [32]. Interestingly, sinapic acid was detected in low proportions only in white grape balsamic and common vinegars (Tables S4 and S5). Nićiforović and Abramovič [33] reported that sinapic acid is one of the phytochemical components of the genus *Vitis* and displays high antioxidant, antimicrobial, anti-inflammatory, anticancer and anti-anxiety activity. The presence of chrysin, quercetin and kaempferol was considerably higher in the common vinegars compared to the balsamic ones. Naringenin was detected only in the samples WW4-WW7 and F5 and may be attributed to their enrichment with oregano, basil, rosemary and thyme as well as sea buckthorn. Apple cider vinegars were characterized by higher proportion of phloretic acid and lower proportion of gallic acid compared to the grape ones. Regarding phenolic aldehydes, only vanillin was detected in low proportion in all common and in most of balsamic vinegars, in accordance with previous findings [34].

### 3.3. Colour Parameters of the Vinegars

Colour is an important factor for consumer acceptance. Red grape balsamic vinegars (RGBV) having the lowest (*p* < 0.05) L* (lightness) and a* values, were the darkest and the greenest in appearance, compared to the all other studied vinegars (Table 4).

The red grape balsamic vinegars with honey (RGBVH) showed higher (*p* < 0.05) L* and b* (yellowness) values compared to the respective ones of RGBV, probably due to their enrichment with honey. Interestingly, balsamic vinegars were characterized by negative a* values, instead of positive a* values for the common vinegars. Li et al. [35] reported that a* and b* values were decreased and increased, respectively, during acetic fermentation of vinegars. Furthermore, white grape and fruit vinegars showed higher (*p* < 0.05) brightness and lower yellowness than the vinegars from red grapes. The colour density is the sum of the absorbance, measured spectrophotometrically, at 420 and 520 nm. High values of colour density denote vinegars with dark or brown red colour, whereas white or light yellow vinegars possess a low colour density (Table 4). The tint value of the vinegar reflects the ratio of yellow to red colouring. Therefore, the white or light yellow vinegars showed higher (*p* < 0.05) tint values than the dark or red ones. In accordance to the aforementioned results, De la Haba et al. [8] reported that lower tint and higher density values indicated that the vinegar has been made from darker wine. Due to the high variations of the colour values among the apple and the other fruit vinegars, results are presented separately (Table 4). Pomegranate and sea buckthorn vinegars exhibited the highest (*p* < 0.05) b* values compared to all the other samples, as well as substantially (*p* < 0.05) higher redness and colour density and lower colour tint, compared to all white or light yellow vinegars. Taking into consideration that pomegranate and sea buckthorn are rich sources of natural anthocyanins [16,36], the aforementioned colour values may reflect higher amounts of anthocyanins in vinegars. Reinforcing this point of view, Cliff et al. [37] reported that the higher anthocyanin content of red wines contributed to the higher colour density and lower colour tint and L* (lightness) values. Hue or hue-angle value, expressed as degrees, defines the category of colour. According to the results of the study, RGBV showed the highest (*p* < 0.05) hue-angle values, followed by RGBVH, pomegranate and sea buckthorn vinegars, all corresponding to the yellow-green region of the colour wheel. Additionally, white grape balsamic, red grape, white grape and apple vinegars exhibited hue-angle values corresponding to the yellow region of the colour wheel. 

High positive Spearman correlation values were observed among the TPC, ABTS and FRAP results and the hue-angle and colour density values (Table 3). Moreover, significant negative correlations were determined among the TPC, ABTS and FRAP results and the L* and a* values. Therefore, an important conclusion drawn by the above results is that the vinegars with high TPC and radical scavenging-antioxidant activity exhibited high hue-angle and colour density values and low L* and a* values. Furthermore, the combined use of colorimetric and spectrophotometric methods may serve as a powerful tool to identify the type of the vinegar and to correlate the colour values with the vinegar compositional quality.

### 3.4. FTIR Vinegar Spectra Interpretation

**Figure 1:**
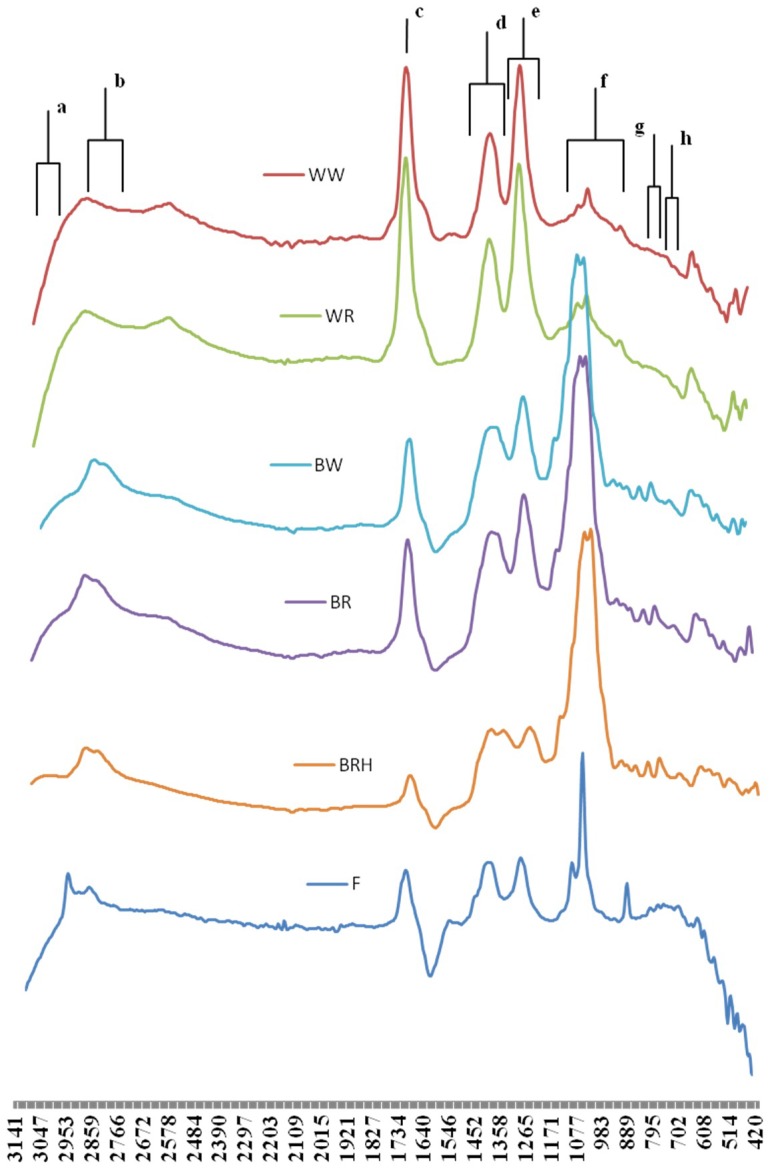
FT-IR absorbance spectra of vinegars. Main absorbance peaks are described using annotations above the upper spectrum: a. 3100–3050 cm^−1^: C–H stretching vibrations of aryl or vinyl groups; b. 2940–2840 cm^−1^: C–H stretching of methyl- and methylene groups of carbohydrates or carboxylic acids; c. 1721 cm^−1^: C=O stretching of carboxylic acids; d. 1425–1380 cm^−1^: C–C stretching vibration of phenyl groups of aromatic compounds; e. 1300–1260 cm^−1^: C–O stretching vibrations of hydroxyflavonoids; f. 1170–950 cm^−1^: C–O and C–C stretching of carbohydrates, polysaccharides, or flavonoids; (broad bands) stretching and bending vibrations of CH_2_OH group of carbohydrates; g. 825–810 cm^−1^: C–H out of plane bending vibrations of phenyl ring of polyphenols; h. 790 to 760 cm^−1^: aromatic ring vibrations.

Fourier Transform Infrared (FT-IR) spectroscopy provides structural information on molecular features of a large range of compounds. Figure 1 represents the comparative FT-IR spectra (3500 to 450 cm^−1^) of the studied vinegars.

According to all spectra, the most characteristic bands in order to assess the vinegars’ composition and any differentiations between them are located from 3100 to 2800 cm^−1^, around the wavenumber of 1700 cm^−1^ and from 1450 to 770 cm^−1^. Most of these bands are associated with organic acids, phenolic compounds and carbohydrates. 

The band from 3100 to 3050 cm^−1^, corresponds to aryl or vinyl C–H stretching vibrations [38], whereas the band from 2940 to 2840 cm^−1^ is assigned to C–H stretching of methyl- and methylene groups of carbohydrates or carboxylic acids [39]. The red grape balsamic vinegars with honey (RGBVH) exhibited the highest (*p* < 0.05) intensities for both the aforementioned bands, followed by red and white grape balsamic vinegars (RGBV, WGBV), while the common vinegars gave the lowest intensities (Table S6). 

Carboxylic acids are characterized by C=O stretching around the wavenumber of 1721 cm^−1^ [40]. Based on our results, RGBVH and FV exhibited lower (*p* < 0.05) intensities at 1721 cm^−1^ compared to those of red vinegars, regardless if they were balsamic or common. Interestingly, this finding was also in accordance with the lower organic acid content of the specific vinegars (Table 5), when compared to red vinegars.

Organic acids in vinegars are probably originating from the wine composition, or the fermentation process [41]. All examined vinegars were found to contain several organic acids such as acetic, malic, citric, tartaric and lactic (Table 5), all being important components that affect the flavour and aroma. Malic acid predominated in all studied vinegars, followed by acetic acid, whereas tartaric acid was found in lower amounts. According to Theron and Lues [42], malic acid content is associated with the wine origin and the oenological techniques and is found in higher amounts in balsamic vinegars. Citric acid content was significantly high in red grape balsamic vinegars with honey, characteristic of honey presence, given the fact that Thymus sp. honeys have high citric acid contents [43]. On the basis of the above results, citric acid could be an indicator of vinegar enrichment with honey. Citric acid was also found in RGBV and FV in modest amounts, probably being formed during the fermentation processes [44]. Finally, lactic acid was identified only in RGBV, RGBVH and FV in low amounts, probably originating during malolactic fermentation [42].

The band at 1425–1380 cm^−1^ is assigned to C–C stretching vibration in phenyl groups of aromatic compounds [45,46] and the band at 1300–1260 cm^−1^ is ascribed to C–O stretching vibrations of hydroxyflavonoids [47]. According to our results (Table S6), balsamic vinegars showed significantly higher intensities in these spectra bands than the common ones; therefore, they could be good indicators for the discrimination among the vinegars. The bands in the region 1170–950 cm^−^ could be assigned to C–O and C–C stretching of carbohydrates, polysaccharides, or flavonoids [48]. Moreover, it is reported [49] that broad bands in the aforementioned region are due to stretching and bending vibrations of CH_2_OH group of carbohydrates originating from the grape sugars. The RGBVH presented significantly (*p* < 0.05) higher intensities (over than 1.5 fold higher) in the region 1080–1040 cm^−1^, compared to the rest red and white grape balsamic vinegars, whereas the RGBV and the WGBV higher (over than 13 fold higher) intensities than the common vinegars (Table S6). This result is attributed to the high sugar content of balsamic vinegars, especially fructose (Table 5), as opposed to common vinegars where no sugars were detected. Moreover, RGBVH showed significantly (*p* < 0.05) high fructose and sucrose content, as expected. 

The peak at 825–810 cm^−1^ could be assigned to C–H out of plane bending vibrations of phenyl ring of polyphenols [50], whereas the band between the wavenumbers from 790 to 770 cm^−1^ could be assigned to aromatic ring vibrations [47]. As these bands showed the highest (*p* < 0.05) signal in RGBVH, followed by RGBV and WGBV (Table S6), the results of FT-IR spectra are consistent with TPC results in balsamic vinegars characterised by high total phenolic content. 

Interestingly, high positive Spearman correlation values were observed among the TPC, ABTS and FRAP results and the intensity values at 1425–1380 cm^−1^ (0.748, 0.753 and 0.822, respectively *p* < 0.01), the intensity values at 1300–1260 cm^−1^ (0.750, 0.756 and 0.819, respectively *p* < 0.01) and the intensity values at 825–770 cm^−1^ (0.685, 0.680 and 0.756, respectively *p* < 0.01). This high correlation confirms that the vibrations in the aforementioned areas are associated with substances (aromatic compounds, polyphenols and hydroxyflavonoids) with significant radical scavenging and antioxidant activity.

**Figure 2:**
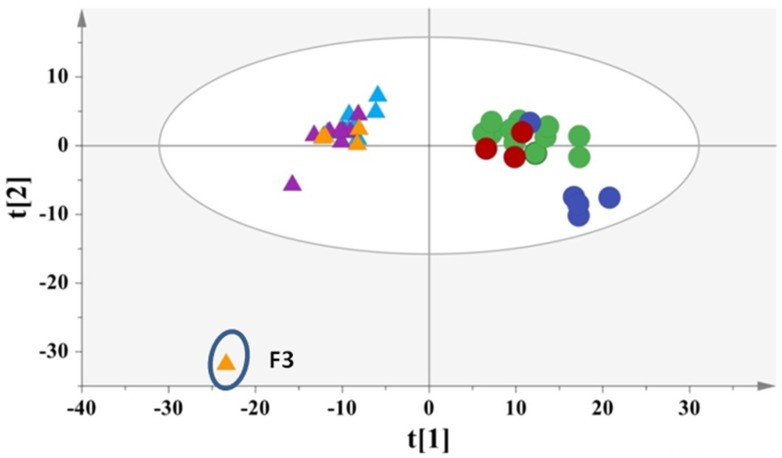
PCA, N = 43, R^2^X(cum)= 0.94, Q^2^(cum)= 0.87 (Circles = balsamic vinegars, Triangles = common vinegars, green = red balsamic vinegars with honey, blue = red balsamic vinegars, red = white balsamic vinegars, turquoise = red common vinegars, purple = white common vinegars, orange = common vinegars from fruits, F3 = common vinegar with Sea Buckthorn).

The extracted PCA model differentiated the samples according to their type (Figure 2). In fact, the balsamic vinegars are discriminated from the common ones along the first principal component. The most characteristic IR fingerprint is ascribed to the balsamic vinegars with honey, as they are localized in the fourth quadrant exhibited decreasing intensity at 1730–1700 cm^−1^ and increasing intensity at 3140–2840 cm^−1^ and 1430–770 cm^−1^ (Figure S1A). The red and white balsamic vinegars shared common IR characteristics such as increasing intensities at 3140-2840 cm^−1^ and 1430–770 cm^−1^ (Figure S1B). Interestingly, the Sea Buckthorn vinegar, shown as an outlier in the statistical model (Figure 2), is characterized by decreasing intensities at 3100–2840 cm^−1^, 1730–1700 cm^−1^, 1430–1260 cm^−1^, 1080–770 cm^−1^ (Figure S1C). Finally, the common vinegars exhibited increasing intensities at 1730–1700 cm^−1^ and decreasing intensities at 3140–2840 cm^−1^, 1430–1260 cm^−1^ and 1080–770 cm^−1^ (Figure S1D).

### 3.5. ^1^H NMR Vinegar Spectra Interpretation

**Figure 3:**
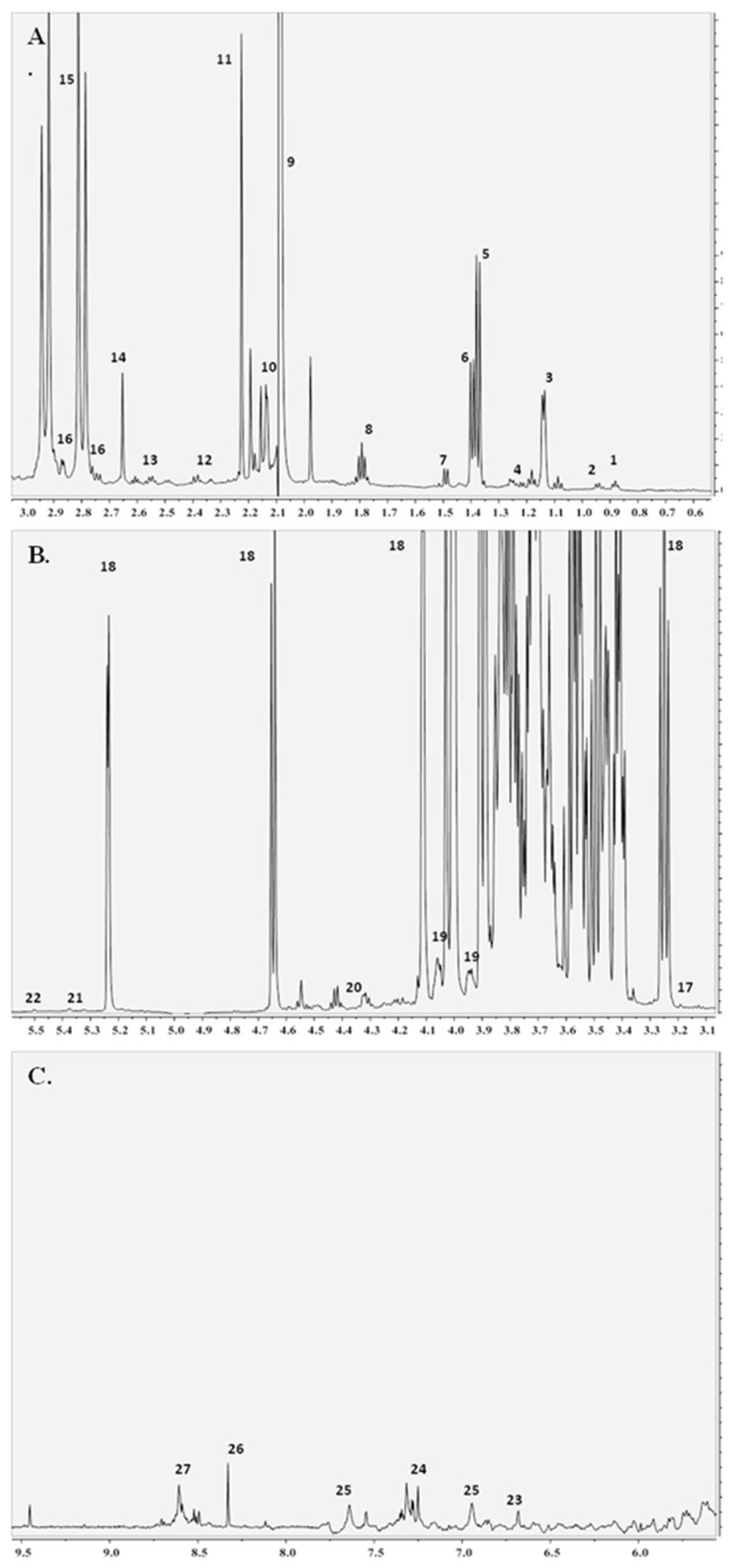
^1^H NMR spectrum of the balsamic vinegar with honey. (**A**) (Peak assignment: 1: leucine, 2: isoleucine, 3: 2,3-butanediol, 4: ethyl acetate, 5: lactic acid, 6: 3-hydroxy-2-butanone, 7: alanine, 8: lysine, 9: acetic acid, 10: acetaldehyde, 11: acetoacetate, 12: glutamine, 13: proline, 14: succinic acid, 15: citric acid, 16: malic acid, (**B**) 17: methanol, 18: glucose, 19: fructose, 20: tartaric acid, 21: sucrose, 22: maltose, (**C**) 23: tyrosol, 24: phenyl alanine, 25: 5-hydroxy methyl furfural, 26: formic acid, 27: furfural).

A representative ^1^H NMR spectrum is presented in Figure 3 for the balsamic vinegar with honey. The assignment of the NMR peaks enabled the identification of 27 metabolites, including mono and oligosaccharides, amino acids as well as other organic acids (acetic, citric, formic, lactic, malic and succinic acids) and phenolic compounds (gallic acid, catechin, epicatechin, chlorogenic acid, caffeic acid and p-coumaric acid), in agreement with previous NMR studies [10,11]. 

The box plots of variations in concentration (presented as integral areas) of identified compounds for the six categories of vinegars are presented in Figure S2. In general, the trends observed were found to be consistent with the IR ones, in respect to the organic acid and sugar content. The box plots hinted a differentiation between balsamic and common vinegars, since increasing values in all compounds were framed for the first. The balsamic vinegars with honey showed the highest concentration values in isoleucine, malic acid, citric acid, tartaric acid, glycerol, glucose, fructose, sucrose, alanine, methanol, lysine, 3-hydroxy-2-butanone and 2,3 butanediol. Balsamic vinegars showed increasing concentration values in lactic acid, valine, succinic acid and pyruvic acid. White balsamic vinegars showed high values in 2,3-butanediol, ethyl acetate, sucrose and the lowest in isoleucine and leucine.

## 4. Conclusions

In the present study, forty three samples of balsamic and common vinegars were examined. Spectrophotometric, chromatographic, colorimetric and spectroscopic methods were synergistically applied in order to assess the vinegar’s compositional profile and to pinpoint the features that influence their type. The strongest antioxidant and radical scavenging capacity was exhibited by the red grape balsamic vinegars; a finding which was also correlated to the highest level of total phenolic content as well as to highest hue-angle and colour density values and lowest lightness and a* values. FT-IR spectra analysis determined specific organic acids and carbohydrates, as well as the occurrence of phenolic compounds. High positive correlation values were observed among the TPC, ABTS and FRAP values and IR intensities at 1425–1380 cm^−1^, 1300–1260 cm^−1^ and 825–770 cm^−1^, mainly attributed to the vibrations of aromatic compounds, polyphenols and hydroxyflavonoids. NMR spectroscopy enabled the identification of 27 metabolites in each type of vinegar. Concentration box plots of characteristic compounds exhibited similar trends with the FT-IR results especially in respect to the organic acid and sugar content.

## Figures and Tables

**Table 1:** Labelling of vinegar samples.

Category	Sample no	Vinegars	Brand Name	Acidity %
RGBV	BR1	Balsamic red	TOP–Minerva	6
	BR2	Balsamic red	Meteora (Agricultural Cooperative of Trikala)	6
	BR3	Balsamic red	Aceto Balamico di Modena from Atlanta S.A.	6
	BR4	Balsamic red	Modena	6
	BR5	Balsamic red	Aceto Balamico di Modena, Villa Lambrusco, Italy	6
	BR6	Balsamic red	Modena–Altis, Unilever	6
	BR7	Balsamic red	AB Vassilopoulos	6
	BR8	Balsamic red	Aceto Balamico di Modena, Villa Trebbiana, Italy	6
	BR9	Balsamic red	Pan	6
	BR10	Balsamic red	Ageing balsamic vinegar Aceto Botanico Adriani Gold Seal from Lazaridi	6
	BR11	Balsamic red Bio	Papadimitriou S.A.	6
	BR12	Balsamic red Bio	AB Vassilopoulos	6
RGBVH	BRH1	Balsamic red with honey	TOP–Minerva	6
	BRH2	Balsamic red with honey	Kaloudi–Angel Foods	6
	BRH3	Balsamic red with honey	AB Vassilopoulos	6
	BRH4	Balsamic red with honey	Pan	6
	BRH5	Balsamic red with honey	Oxymelo–Gaea	6
WGBV	BW1	Balsamic white	Papadimitriou S.A.	6
	BW2	Balsamic white	Contimento Bianco–Atlanta S.A.	5.4
	BW3	Balsamic white	Villa Grimelli, Italy	5.4
RGV	WR1	Wine red	TOP–Minerva	6
	WR2	Wine red	Pure vinegar from red wine from Galaxias	7
	WR3	Wine red	Fino–HAINA Greek Vinegar	6
	WR4	Wine red	Pan	8
	WR5	Wine red	Meteora (Agricultural Cooperative of Trikala)	6
	WR6	Wine red	Paros–Sifnaios K. & Co	6
	WR7	Wine red	AB Vassilopoulos	7
	WR8	Wine red	Bio red vinegar from Papadimitriou S.A.	6
	WR9	Wine red	Tripodakis	6
	WR10	Wine red	Kriteli–Union of agricultural cooperatives of Heraklion	6
WGV	WW1	Wine white	TOP–Minerva	7
	WW2	Wine white	Fino–HAINA Greek Vinegar	6
	WW3	Wine white	Meteora (Agricultural Cooperative of Trikala)	6
	WW4	Wine white	Paros–Sifnaios K. & Co	6
	WW5	Wine white	MESSINO OREGANO Papadeas D. & Co	6
	WW6	Wine white	MESSINO BASIL Papadeas D. & Co	6
	WW7	Wine white with rosemary & thyme	Pan	6
	WW8	Champagne wine	La Marne–Champagne Ardenne Vinegar Charbonneaux Brabant S.A.	7
FV	F1	Apple	TOP–Minerva	6
	F2	Apple	Paros–Sifnaios K. & Co	6
	F3	Apple	Olympos–Viofresko	6
	F4	Pomegranate	Ayanoglou S.A.	6
	F5	Sea Buckthorn	Berryland	6

**Table 2:** Descriptive statistics of total phenolic content (TPC) and radical scavenging -antioxidant activity of vinegars.

Vinegars	Mean	S.D.	Median	S.E.M.	Minimum	Maximum
	**Total phenolic content (TPC) as mg of gallic acid equivalents/L**					
RGBV ^a^	1556.86a	586.81	1418.50	169.39	853.50	2867.33
RGBVH ^b^	721.85b	103.62	734.00	46.34	560.70	847.33
WGBV ^c^	145.85c	8.38	146.83	4.84	137.03	153.70
RGV ^d^	239.32d	77.90	227.73	24.63	134.70	382.73
WGV ^e^	109.04c	56.95	116.65	20.13	29.75	185.75
FV ^f^	139.12c	164.18	82.70	73.42	17.88	428.37
	**Radical scavenging activity as mg of trolox equivalents/L**					
RGBV ^a^	1862.84a	981.84	1557.79	283.43	1014.41	4417.44
RGBVH ^b^	1055.21a	164.30	980.39	73.47	908.56	1267.11
WGBV ^c^	187.97c	106.80	143.59	61.66	110.52	309.82
RGV ^d^	554.30d	230.50	542.18	72.89	247.16	966.38
WGV ^e^	135.59c	96.16	99.12	33.99	30.63	305.03
FV ^f^	190.34c	184.32	127.93	82.43	46.12	496.49
	**Antioxidant activity as mg of FeSO_4_·7H_2_O equivalents/L**					
RGBV ^a^	13897.40a	5663.38	11971.62	1634.87	7689.42	26,293.23
RGBVH ^b^	9060.96a	3313.93	9921.26	1482.04	3635.04	11,674.30
WGBV ^c^	2102.64b	1025.68	1571.32	592.18	1451.62	3284.97
RGV ^d^	3356.99b	1205.68	3090.74	381.27	1994.42	5497.76
WGV ^e^	947.16c	528.34	836.00	186.79	292.31	1873.95
FV ^f^	946.45c	830.57	652.59	371.44	350.50	2409.87

^a^ RGBV: red grape balsamic vinegars (*n* = 12), ^b^ RGBVH: red grape balsamic vinegars with honey (*n* = 5), ^c^ WGBV: white grape balsamic vinegars (*n* = 3), ^d^ RGV: red grape vinegars (*n* = 10), ^e^ WGV: white grape vinegars (*n* = 8), ^f^ FV: fruit vinegars (*n* = 5). Means in the same column bearing different letters differ significantly (*p* < 0.05).

**Table 3:** Spearman correlation among the spectrophotometric assays Folin–Ciocalteu (TPC), ABTS and FRAP and colour parameters (L*, a*, b*, h, CD, CT).

Variables	ABTS	FRAP	L*	a*	b*	h	CD	CT
TPC	0.980	0.960	−0.874	−0.750	0.344	0.916	0.918	−0.009
ABTS		0.983	−0.847	-0.698	0.390	0.889	0.909	−0.022
FRAP			−0.842	−0.711	0.392	0.872	0.892	0.050
L*				0.781	−0.413	−0.902	−0.873	−0.016
a*					−0.016	−0.774	−0.719	−0.474
b*						0.473	0.496	−0.260
h							0.947	0.025
CD								−0.017

Correlation is significant at the 0.01 level (2-tailed).

**Table 4:** Colour parameters of vinegars.

Vinegars	L*	a*	b*	h	CD	CT
RGBV ^a^	12.00 ± 0.92a	−0.94 ± 0.16a	3.07 ± 0.30a	106.86 ± 4.50a	35.42 ± 11.46a	2.68 ± 0.35a
RGBVH ^b^	13.83 ± 0.33b	−0.54 ± 0.09b	3.86 ± 0.17b	90.78 ± 11.29b	18.49 ± 3.18b	2.72 ± 0.36a
WGBV ^c^	16.90 ± 0.78c	−0.21 ± 0.03c	1.87 ± 0.17c	72.09 ± 4.07c	0.73 ± 0.05c	5.64 ± 0.87b
RGV ^d^	16.79 ± 0.81c	1.13 ± 0.32d	3.73 ± 0.78ab	77.92 ± 6.97c	0.95 ± 0.12d	1.98 ± 0.34c
WGV ^e^	18.44 ± 0.53d	0.48 ± 0.09e	1.42 ± 0.27d	63.41 ± 5.42d	0.29 ± 0.02e	3.57 ± 0.59d
F1-F3 ^f^	18.52 ± 0.37d	0.54 ± 0.03e	1.39 ± 0.24d	64.08 ± 5.16d	0.50 ± 0.02f	3.87 ± 0.36d
F4-F5 ^g^	17.34 ± 1.40cd	1.66 ± 0.53d	4.94 ± 0.46e	80.29 ± 3.65b	2.49 ± 0.34g	1.52 ± 0.62c

^a^ RGBV: red grape balsamic vinegars (*n* = 12), ^b^ RGBVH: red grape balsamic vinegars with honey (*n* = 5), ^c^ WGBV: white grape balsamic vinegars (*n* = 3), ^d^ RGV: red grape vinegars (*n* = 10), ^e^ WGV: white grape vinegars (*n* = 8), ^f^ F1–F3: apple vinegars (*n* = 3), ^g^ F4–F5: Pomegranate and Sea Buckthorn vinegars. Means in the same column bearing different letters differ significantly (*p* < 0.05).

**Table 5:** Vinegars’ organic acid and sugar content.

Sample	Ethanol (% vol)	Acetic acid (g/L)	Malic acid (g/L)	Citric acid (g/L)	Lactic acid (g/L)	Tartaric acid (g/L)	Glycerol (g/L)	Fructose (g/L)	Glucose (g/L)	Sucrose (g/L)
RGBV ^a^	0.49 ± 0.06a	20.16 ± 0.60a	39.02 ± 1.12a	0.77 ± 0.27a	0.07 ± 0.01a	7.37 ± 1.83a	4.37 ± 2.14	113.44 ± 9.15a	34.04 ± 0.24a	0.94 ± 0.26a
RGBVH ^b^	0.69 ± 0.08b	14.01 ± 2.63b	25.63 ± 1.28b	7.56 ± 1.24b	0.22 ± 0.02b	4.06 ± 0.52b	3.52 ± 2.85	170.21 ± 18.95b	28.12 ± 0.46b	7.05 ± 2.32b
WGBV ^c^	0.53 ± 0.05a	20.58 ± 0.40a	37.54 ± 1.02a	-	-	7.98 ± 1.10a	4.31 ± 1.30	96.39 ± 5.34c	7.79 ± 0.11c	1.06 ± 0.64a
RGV ^d^	0.72 ± 0.09b	21.78 ± 0.81c	41.39 ± 2.20c	-	-	5.91 ± 0.79c	1.20 ± 1.78	-	-	-
WGV ^e^	0.69 ± 0.11b	19.71 ± 0.93a	38.04 ± 1.62a	-	-	5.25 ± 0.86c	0.45 ± 0.67	-	-	-
FV ^f^	1.21 ± 0.27c	11.97 ± 4.80d	22.87 ± 5.41b	1.80 ± 1.16a	0.12 ± 0.02c	3.17 ± 1.07d	1.78 ± 2.35	0.09 ± 0.07d	-	-

^a^ RGBV: red grape balsamic vinegars (*n* = 12), ^b^ RGBVH: red grape balsamic vinegars with honey (*n* = 5), ^c^ WGBV: white grape balsamic vinegars (*n* = 3), ^d^ RGV: red grape vinegars (*n* = 10), ^e^ WGV: white grape vinegars (*n* = 8), ^f^ FV: fruit vinegars (*n* = 5). Means in the same column bearing different letters differ significantly (*p* < 0.05).

## Supplementary Materials

The following are available online at http://www.mdpi.com/2076-3921/7/10/139/s1, Table S1: Target and qualifier ions for the trimethylsilyl ethers (TMS) of phenolic compounds and the internal standard (IS), Table S2: Total phenolic content, radical scavenging and antioxidant activity of balsamic vinegars samples, Table S3: Total phenolic content, radical scavenging and antioxidant activity of common vinegars samples, Table S4: Major phenolic compounds of the balsamic vinegars (% of total quantified phenols), Table S5: Major phenolic compounds of the common vinegars (% of total quantified phenols), Table S6: Spectra bands intensities of vinegars, Figure S1: Contribution plots extracted from the PCA model. A. Contribution plot of the balsamic vinegars with honey, B. Contribution plot of the red and white balsamic vinegars, C. Contribution plot of the vinegar with the embedded Sea Buckthorn, D. Contribution plot of the common vinegars, Figure S2: Box plots of the significant metabolites as pinpointed by Anova on the NMR data (BRH= balsamic vinegars with honey, BR= red balsamic vinegars, BW= white balsamic vinegars, WR= red common vinegars, WW= white common vinegars, F= common vinegars from fruits).

## References

[1] Budak N.H., Aykin E., Seydim A.C., Greene A.K., Guzel-Seydim Z.B. (2014). Functional properties of vinegar. J. Food Sci..

[2] Nakamura K., Ogasawara Y., Endou K., Fujimori S., Koyama M., Akana H. (2010). Phenolic compounds responsible for the superoxide dismutase-like activity in High-Brix apple vinegar. J. Agric. Food Chem..

[3] Chen H., Chen T., Giudici P., Chen F. (2016). Vinegar functions on health: Constituents, sources, and formation mechanisms. Compr. Rev. Food Sci. Food Saf..

[4] Nazıroğlu M., Güler M., Özgül C., Saydam G., Küçükayaz M., Sözbir E. (2014). Apple cider vinegar modulates serum lipid profile, erythrocyte, kidney, and liver membrane oxidative stress in ovariectomized mice fed high cholesterol. J. Membr. Biol..

[5] Wu D., Kimura F., Takashima A., Shimizu Y., Takebayashi A., Kita N., Zhang G., Murakami T. (2013). Intake of vinegar beverage is associated with restoration of ovulatory function in women with polycystic ovary syndrome. Tohoku J. Exp. Med..

[6] Kelebek H., Kadiroğlu P., Demircan N.B., Selli S. (2017). Screening of bioactive components in grape and apple vinegars: Antioxidant and antimicrobial potential. J. Inst. Brew..

[7] Torrecilla J.S., Aroca-Santos R., Cancilla J.C., Matute G. (2016). Linear and non-linear modeling to identify vinegars in blends through spectroscopic data. LWT-Food Sci. Technol..

[8] De la Haba M.-J., Arias M., Ramírez P., Lopez M.-I., Sanchez M.-T. (2014). Characterizing and authenticating Montilla-Moriles PDO vinegars using near infrared reflectance spectroscopy (*NIRS*) technology. Sensors.

[9] Callejón R.M., Amigo J.M., Pairo E., Garmón S., Ocaña J.A., Morales M.L. (2012). Classification of sherry vinegars by combining multidimensional fluorescence, parafac and different classification approaches. Talanta.

[10] Wang X., Wang J., Kamal G.M., Jiang B., Sun P., Zhang X., Liu M. (2016). Characterization and comparison of commercial Chinese cereal and European grape vinegars using ^1^H NMR spectroscopy combined with multivariate analysis. Chin. J. Chem..

[11] Boffo E.F., Tavares L.A., Ferreira M.M., Ferreira A.G. (2009). Classification of Brazilian vinegars according to their ^1^H NMR spectra by pattern recognition analysis. LWT-Food Sci. Technol..

[12] Consonni R., Cagliani L.R., Benevelli F., Spraul M., Humpfer E., Stocchero M. (2008). NMR and chemometric methods: A powerful combination for characterization of balsamic and traditional balsamic vinegar of Modena. Anal. Chim. Acta.

[13] Grégrová A., Čížková H., Mazáč J., Voldřich M. (2012). Hodnocení autenticity kvasného lihového octa (část II): Analýza vzorků z tržní sítě Authenticity Assessment of Spirit Vinegar (*Part II*): Analysis of Samples from Distribution Chain. Kvasny Prum..

[14] Yu Y.J., Lu Z.M., Yu N.H., Xu W., Li G.Q., Shi J.S., Xu Z.H. (2012). HS-SPME/GC-MS and chemometrics for volatile composition of Chinese traditional aromatic vinegar in the Zhenjiang region. J. Inst. Brew..

[15] Andreou V., Strati I.F., Fotakis C., Liouni M., Zoumpoulakis P., Sinanoglou V.J. (2018). Herbal distillates: A new era of grape marc distillates with enriched antioxidant profile. Food Chem..

[16] Lantzouraki D.Z., Sinanoglou V.J., Zoumpoulakis P.G., Glamoclija J., Ciric A., Sokovic M., Heropoulos G., Proestos C. (2015). Antiradical–antimicrobial activity and phenolic profile of pomegranate (*Punica granatum* L.) juices from different cultivars: A comparative study. RSC. Adv..

[17] Lantzouraki D.Z., Sinanoglou V.J., Zoumpoulakis P., Proestos C. (2016). Comparison of the Antioxidant and Antiradical Activity of Pomegranate (*Punica granatum* L.) by Ultrasound-Assisted and Classical Extraction. Anal. Lett..

[18] Soleas G., Diamandis E., Karumanchiri A., Goldberg D. (1997). A multiresidue derivatization gas chromatographic assay for fifteen phenolic constituents with mass selective detection. Anal. Chem..

[19] Kaliora A.C., Kogiannou D.A.A., Kefalas P., Papassideri I.S., Kalogeropoulos N. (2014). Phenolic profiles and antioxidant and anticarcinogenic activities of Greek herbal infusions; balancing delight and chemoprevention. Food Chem..

[20] Tomasi G., Van Den Berg F., Andersson C. (2004). Correlation optimized warping and dynamic time warping as preprocessing methods for chromatographic data. J. Chemom..

[21] Trygg J., Holmes E., Lundstedt T. (2007). Chemometrics in metabonomics. J. Proteome Res..

[22] Eriksson L., Johansson E., Kettaneh-Wold N., Trygg J., Wikström C., Wold S. (2006). Multi- and Megavariate Data Analysis.

[23] Giudici P., Gullo M., Solieri L., Falcone P.M. (2009). Technological and microbiological aspects of traditional balsamic vinegar and their influence on quality and sensorial properties. Advances in Food and Nutrition Research.

[24] Tesfaye W., Morales M.L., Garcıa-Parrilla M.C., Troncoso A.M. (2002). Wine vinegar: Technology, authenticity and quality evaluation. Trends Food Sci. Technol..

[25] Almaraz-Abarca N., Campos M.D.G., Ávila-Reyes J.A., Naranjo-Jiménez N., Herrera-Corral J., González-Valdez L.S. (2004). Variability of antioxidant activity among honeybee-collected pollen of different botanical origin. Interciencia.

[26] Negi B., Dey G. (2009). Comparative analysis of total phenolic content in sea buckthorn wine and other selected fruit wines. World Acad. Sci. Eng. Technol..

[27] Eccleston C., Baoru Y., Tahvonen R., Kallio H., Rimbach G.H., Minihane A.M. (2002). Effects of an antioxidant-rich juice (*sea buckthorn*) on risk factors for coronary heart disease in humans. J. Nutr. Biochem..

[28] Sakanaka S., Ishihara Y. (2008). Comparison of antioxidant properties of persimmon vinegar and some other commercial vinegars in radical scavenging assays and on lipid oxidation in tuna homogenates. Food Chem..

[29] Dávalos A., Bartolomé B., Gómez-Cordovés C. (2005). Antioxidant properties of commercial grape juices and vinegars. Food Chem..

[30] Ozturk I., Caliskan O.Z.N.U.R., Tornuk F., Ozcan N., Yalcin H., Baslar M., Sagdic O. (2015). Antioxidant, antimicrobial, mineral, volatile, physicochemical and microbiological characteristics of traditional home-made Turkish vinegars. LWT-Food Sci. Technol..

[31] Gil-Muñoz R. (1999). Evolution of phenolic compounds during wine fermentation and post-fermentation: Influence of grape temperature. J. Food Compos. Anal..

[32] Swiegers J.H., Bartowsky E.J., Henschke P.A., Pretorius I.S. (2005). Yeast and bacterial modulation of wine aroma and flavor. Aust. J. Grape. Wine Res..

[33] Nićiforović N., Abramovič H. (2014). Sinapic acid and its derivatives: Natural sources and bioactivity. Compr. Rev. Food Sci. Food Saf..

[34] Barnaba C., Dellacassa E., Nicolini G., Nardin T., Malacarne M., Larcher R. (2015). Identification and quantification of 56 targeted phenols in wines, spirits, and vinegars by online solid-phase extraction–ultrahigh-performance liquid chromatography–quadrupole–orbitrap mass spectrometry. J. Chromatogr. A.

[35] Li T., Lo Y.M., Moon B. (2014). Feasibility of using Hericium erinaceus as the substrate for vinegar fermentation. LWT-Food Sci. Technol..

[36] Yildiz H., Sengul M., Celik F., Ercisli S., Duralija B. (2012). Bioactive content of Sea Buckthorn (*Hippophae rhamnoides L*.) berries from Turkey. Agric. Conspec. Sci. (ACS).

[37] Cliff M.A., King M.C., Schlosser J. (2007). Anthocyanin, phenolic composition, colour measurement and sensory analysis of BC commercial red wines. Food Res. Int..

[38] Smith B.C. (2016). Distinguishing Structural Isomers: Mono-and Disubstituted Benzene Rings. Spectroscopy.

[39] Domínguez-Martínez I., Meza-Márquez O.G., Osorio-Revilla G., Proal-Nájera J., Gallardo-Velázquez T. (2014). Determination of capsaicin, ascorbic acid, total phenolic compounds and antioxidant activity of capsicum annuum l. var. serrano by mid infrared spectroscopy (Mid-FTIR) and chemometric analysis. J Korean. Soc. Appl. Biol. Chem..

[40] Ríos-Reina R., Callejón R.M., Oliver-Pozo C., Amigo J.M., García-González D.L. (2017). ATR-FTIR as a potential tool for controlling high quality vinegar categories. Food Control.

[41] Sanarico D., Motta S., Bertolini L., Antonelli A. (2003). HPLC determination of organic acids in traditional balsamic vinegar of Reggio Emilia. J. Liq. Chromatogr. Relat. Technol..

[42] Theron M.M., Lues J.R. (2010). Incidental and natural organic acid occurrence. Organic Acids and Food Preservation.

[43] Mato I., Huidobro J.F., Simal-Lozano J., Sancho M.T. (2003). Significance of nonaromatic organic acids in honey. J. Food Prot..

[44] Aguiar A., Nascimento R.A.A., Ferretti L.P., Gonçalves A.R. (2005). Determination of organic acids and ethanol in commercial vinegars. Braz. J. Food Technol..

[45] Murugesh S., Vino P. (2017). Phytochemical constituents, antioxidant activity and FT-IR analysis of *Pisonia grandis* leaf extracts. Int. J. Pharmacogn. Phytochem. Res..

[46] Baciu A., Ranga F., Fetea F., Zavoi S., Socaciu C. (2013). Fingerprinting food supplements and their botanical ingredients by coupled UV/Vis/FTIR spectrometry. Bulletin of university of agricultural sciences and veterinary medicine Cluj-Napoca. Food Sci. Technol..

[47] Oliveira R.N., Mancini M.C., Oliveira F.C.S.D., Passos T.M., Quilty B., Thiré R.M.D.S.M., McGuinness G.B. (2016). FTIR analysis and quantification of phenols and flavonoids of five commercially available plants extracts used in wound healing. Matéria (Rio. De. Janeiro).

[48] Pop R.M., Buzoianu A.D., Raţi I.V., Socaciu C. (2014). Untargeted metabolomics for Sea buckthorn (*Hippophae rhamnoides* ssp. carpatica) berries and leaves: Fourier transform infrared spectroscopy as a rapid approach for Evaluation and discrimination. Not. Bot. Horti AgroboT..

[49] Grassi S., Amigo J.M., Lyndgaard C.B., Foschino R., Casiraghi E. (2014). Beer fermentation: Monitoring of process parameters by FT-NIR and multivariate data analysis. Food Chem..

[50] Schulz H., Baranska M. (2007). Identification and quantification of valuable plant substances by IR and Raman spectroscopy. Vib. Spectrosc..

