# Supplementary material for: On the Characterization and Correlation of Compositional, Antioxidant and Colour Profile of Common and Balsamic Vinegars
Source: Antioxidants (Basel). 2018 Oct 11;7(10):139. doi: 10.3390/antiox7100139 (PMC6210356; doi:10.3390/antiox7100139)
Supplement: Supplementary file 1 [file antioxidants-07-00139-s001.pdf]

Table S1. Target and qualifier ions for the trimethylsilyl ethers (TMS) of phenolic compounds and the internal standard (IS).

Compound	Target ions (*m*/*z*)	Qualifier ions (*m*/*z*)
Vanillin	194	209
Cinnamic acid	205	220
Tyrosol	179	267,282
p-Hydroxy-benzoic acid	267	223,193
p-Hydroxy-phenylacetic acid	252	296,281
3-(4-hydroxyphenyl)-1-propanol (I.S.)	206	191,179
Phloretic acid	192	310
Vanillic acid	297	267,312
Homovanillic alcohol	326	267,311
Protocatechuic acid	193	355,370
3-4 Di-hydroxy-phenylacetic acid	384	267,179
Syringic acid	327	342,312
o-Coumaric acid	293	308,147
p-Coumaric acid	308	293,219
Gallic acid	281	458,443
Ferulic acid	338	323,308
Caffeic acid	396	219,381
Sinapic acid	368	353,338
Resveratrol	444	445,443
Chrysin	383	355,474
Epicatechin	368	355,474
Naringenin	473	296
Catechin	368	355,474
Kaempferol	559	560
Quercetin	647	559,575

Table S2. Total phenolic content, radical scavenging and antioxidant activity of balsamic vinegars samples.

	mg Gallic acid E /L	mg Trolox E /L	mg $FeSO_4 \cdot 7H_2O$/ L
BR1	1268.50 ± 22.91	1469.95 ± 5.22	11299.19 ± 150.74
BR2	1251.83 ± 20.21	1216.64 ± 9.04	11936.87 ± 210.22
BR3	853.50 ± 27.84	1014.41 ± 36.68	7689.42 ± 162.18
BR4	1733.50 ± 10.00	1897.19 ± 13.59	12307.55 ± 167.60
BR5	1660.67 ± 20.00	1958.11 ± 26.11	17512.60 ± 128.99
BR6	2867.33 ± 61.10	4417.44 ± 52.22	26293.23 ± 128.99
BR7	1355.17 ± 43.68	1187.05 ± 28.76	11936.87 ± 58.30
BR8	1647.33 ± 50.33	1524.72 ± 46.41	10647.19 ± 173.89
BR9	1220.17 ± 22.55	1797.17 ± 31.47	14160.98 ± 127.60
BR10	2434.00 ± 46.19	3133.46 ± 34.54	22949.33 ± 157.70
BR11	1481.83 ± 45.09	1590.86 ± 18.34	12006.37 ± 141.56
BR12	908.50 ± 13.23	1147.02 ± 19.77	8029.22 ± 197.04
BRH1	560.70 ± 6.56	925.97 ± 30.60	3635.04 ± 106.17
BRH2	753.70 ± 10.00	980.39 ± 22.76	9921.26 ± 70.78
BRH3	734.00 ± 40.00	1194.01 ± 13.14	11620.24 ± 116.61
BRH4	713.50 ± 35.00	908.56 ± 32.61	8453.96 ± 106.17
BRH5	847.33 ± 61.10	1267.11 ± 18.34	11674.30 ± 167.07

BW1	146.83 ± 12.58	309.82 ± 25.76	3284.97 ± 37.29
BW2	153.70 ± 6.56	143.59 ± 1.38	1571.32 ± 43.12
BW3	137.03 ± 5.51	110.52 ± 5.23	1451.62 ± 15.77

Table S3. Total phenolic content, radical scavenging and antioxidant activity of common vinegars samples.

	**mg Gallic acid E /L**	**mg Trolox E /L**	**mg $FeSO_4{\cdot}7H_2O$/ L**
WR1	218.73 ± 13.32	618.76 ± 25.71	3320.09 ± 160.53
WR2	382.73 ± 12.22	966.38 ± 27.67	5497.76 ± 69.55
WR3	134.70 ± 5.57	247.16 ± 5.28	1994.42 ± 87.57
WR4	350.73 ± 15.01	849.93 ± 57.75	5286.17 ± 134.85
WR5	184.70 ± 2.00	337.23 ± 4.20	2765.87 ± 46.51
WR6	268.07 ± 2.31	647.16 ± 15.95	3534.77 ± 34.98
WR7	236.73 ± 12.06	607.45 ± 25.62	3667.59 ± 111.23
WR8	170.07 ± 1.15	458.55 ± 31.15	2861.39 ± 95.11
WR9	247.37 ± 2.31	476.91 ± 2.72	2161.99 ± 9.36
WR10	199.40 ± 13.11	333.48 ± 76.44	2479.91 ± 70.32
WW1	185.75 ± 2.41	177.54 ± 12.45	867.75 ± 52.69
WW2	61.61 ± 2.27	60.05 ± 3.13	395.90 ± 35.31
WW3	29.75 ± 5.37	30.63 ± 8.76	92.31 ± 6.03
WW4	94.28 ± 2.62	81.63 ± 1.83	802.09 ± 79.39
WW5	54.03 ± 8.50	74.84 ± 15.68	804.25 ± 23.15
WW6	161.48 ± 2.00	238.45 ± 4.58	1500.20 ± 93.66
WW7	146.37 ± 2.52	305.03 ± 11.10	1873.95 ± 14.47
WW8	139.03 ± 1.53	116.62 ± 2.63	1040.86 ± 14.87
F1	82.70 ± 5.29	127.93 ± 5.98	736.47 ± 36.41
F2	88.37 ± 6.43	61.44 ± 5.36	652.59 ± 31.76
F3	78.28 ± 0.80	219.74 ± 19.24	582.80 ± 19.29
F4	17.88 ± 1.20	46.12 ± 1.68	350.50 ± 6.70
F5	428.37 ± 10.79	496.49 ± 13.84	2409.87 ± 154.55

Table S4. Major phenolic compounds of the balsamic vinegars (% of total quantified phenols).

Compounds	BR1	BR2	BR3	BR4	BR5	BR6	BR7	BR8	BR9	BR10	BR11	BR12	BRH1	BRH2	BRH3	BRH4	BRH5	BW1	BW2	BW3
**Phenolic alcohol**																				
Homovanillyl alcohol	0.29	0.09	0.11	0.11	-	0.04	0.05	-	-	0.12	-	-	0.18	-	0.45	0.09	0.43	0.55	1.13	0.97
Tyrosol	6.98	1.97	2.33	1.84	0.60	0.54	0.96	1.12	7.16	1.19	2.11	5.75	2.82	3.51	1.53	1.24	0.78	6.09	9.19	8.25
**Phenolic acids**																				
Caffeic acid	9.28	10.17	8.78	7.52	9.14	6.07	7.42	7.52	9.48	9.38	7.80	6.37	8.86	8.62	9.30	11.01	7.49	22.95	2.19	3.20
Cinnamic acid	0.16	-	0.41	-	0.06	-	0.05	0.10	1.17	0.13	1.08	3.08	0.18	0.28	0.87	0.07	0.17	0.75	0.46	0.57
p-Coumaric acid	2.58	2.09	4.39	1.55	1.92	2.65	1.85	1.87	2.18	2.70	2.04	13.28	1.65	1.04	3.57	1.45	3.21	3.61	0.72	1.33
3,4-Dihydroxyphenylacetic acid	-	0.25	0.10	-	0.07	-	-	-	0.52	0.15	-	-	-	0.31	-	0.10	-	-	-	-
Ferulic acid	0.29	0.20	0.21	0.26	0.18	0.13	0.26	0.16	0.44	0.19	0.59	0.27	0.19	0.37	0.46	0.28	0.36	0.51	0.14	0.10
Gallic acid	65.33	80.48	71.95	69.70	82.76	87.28	78.81	66.96	37.01	82.01	8.16	44.31	75.50	25.27	70.06	70.32	78.59	43.19	66.78	71.39
p-Hydroxybenzoic acid	1.43	0.43	0.84	0.33	0.37	0.26	0.36	0.32	2.17	0.39	1.34	1.28	1.11	0.83	1.28	0.39	0.83	2.71	0.67	0.57
4-Hydroxyphenylacetic acid	0.41	0.36	0.33	0.42	0.09	0.04	0.30	-	1.14	0.17	-	7.21	-	0.50	0.77	0.14	0.33	1.40	1.86	1.21
Phloretic acid	6.70	1.12	1.96	1.44	0.71	0.21	0.21	1.00	5.96	0.25	-	0.10	2.85	2.26	1.79	0.53	0.50	3.83	2.17	2.22
Protocatechuic acid	2.08	1.45	4.82	1.07	3.12	1.21	2.76	1.81	7.66	1.81	4.59	7.28	1.80	1.98	7.30	0.72	5.40	9.74	0.95	2.01
Sinapic acid	-	-	-	-	-	-	-	-	-	-	-	-	-	-	-	-	-	0.38	0.31	0.45
Syringic acid	3.64	0.82	2.74	1.57	0.73	0.38	0.56	6.67	1.31	1.13	22.46	1.63	4.30	6.93	1.80	1.10	1.47	1.28	-	-
Vanillic acid	0.81	0.58	0.67	0.27	0.19	0.12	0.22	0.12	1.72	0.31	2.19	0.39	0.39	0.95	0.83	0.27	0.45	3.02	0.05	0.09
**Phenolic aldehyde**																				
Vanillin	-	-	0.20	0.06	0.06	-	0.06	0.17	0.18	0.05	0.68	-	0.18	0.11	-	0.08	-	-	0.05	0.30
**Flavan-3-ols**																				
Catechin	-	-	-	0.82	-	-	-	-	-	-	-	-	-	-	-	-	-	-	-	-
**Flavones**																				
Chrysin	-	-	-	-	-	1.08	-	-	21.77	-	46.95	-	-	19.80	-	-	-	-	13.33	-
**Flavonols**																				
Quercetin	-	-	-	13.04	-	-	6.14	-	-	-	-	-	-	27.25	-	12.20	-	-	-	-
Kaempferol	-	-	-	-	-	-	-	12.18	-	-	-	9.03	-	-	-	-	-	-	-	7.35
**Stilbenoid**																				
Resveratrol	-	-	0.15	-	-	-	-	-	0.14	-	-	-	-	-	-	-	-	-	-	-

Table S5. Major phenolic compounds of the common vinegars (% of total quantified phenols).

Compounds	WR1	WR2	WR3	WR4	WR5	WR6	WR7	WR8	WR9	WR10	WW1	WW2	WW3	WW4	WW5	WW6	WW7	WW8	F1	F2	F3	F4	F5
**Phenolic alcohol**																							
Homovanillyl alcohol	0.14	0.15	0.89	0.13	0.80	0.24	0.02	0.39	-	0.19	0.10	-	-	0.37	0.06	0.37	0.21	0.13	0.64	0.31	0.49	0.33	0.03
Tyrosol	3.64	5.26	13.86	4.35	9.45	4.50	0.78	17.16	2.11	4.64	1.84	19.17	11.90	7.98	2.15	7.98	4.35	4.70	16.50	6.48	13.27	0.05	0.89
**Phenolic acids**																							
Caffeic acid	8.66	10.21	6.86	10.81	3.95	2.35	4.44	17.82	1.25	2.69	4.04	4.49	1.37	1.77	9.48	1.77	35.00	8.25	3.49	1.60	0.95	2.12	1.17
Cinnamic acid	0.02	0.35	0.13	0.09	0.27	0.08	0.02	0.08	0.04	0.02	-	0.67	3.79	0.12	0.10	0.12	0.13	0.11	0.60	0.06	0.23	1.05	0.01
o-Coumaric acid	1.21	-	1.44	-	1.34	-	0.11	7.37	0.21	0.42	0.23	-	-	-	-	-	2.59	1.96	-	-	0.63	-	2.40
p-Coumaric acid	1.53	1.04	3.72	0.95	1.91	1.45	0.50	4.46	0.31	0.59	0.98	1.62	2.14	0.85	0.52	0.85	2.22	1.31	0.51	0.52	0.56	1.43	0.64
3,4-Dihydroxyphenylacetic acid	0.09	0.52	-	0.49	-	0.07	0.01	0.13	0.06	0.06	0.06	-	-	-	2.08	-	1.42	0.07	-	-	-	-	0.08
Ferulic acid	0.16	0.22	0.36	0.20	0.94	0.17	0.03	0.59	0.06	0.06	0.16	2.45	0.27	0.18	0.09	0.18	1.16	0.20	0.25	0.05	-	0.16	0.06
Gallic acid	63.71	47.20	19.34	51.19	25.84	53.85	90.29	17.52	65.34	64.97	77.86	19.78	10.40	48.31	7.45	48.31	9.53	69.91	7.17	16.12	1.11	91.56	52.30
p-Hydroxybenzoic acid	0.77	1.08	8.91	0.98	5.87	1.30	0.09	5.33	0.90	0.85	0.81	5.79	9.36	3.48	0.77	3.48	1.74	1.18	10.48	4.11	6.38	0.66	0.37
4-Hydroxyphenylacetic acid	0.23	0.88	20.87	0.55	2.11	0.51	0.22	4.37	0.36	1.35	0.23	19.01	2.22	1.09	0.59	1.09	0.91	3.90	6.15	2.55	7.13	0.23	0.41
Phloretic acid	3.96	3.39	4.14	3.30	7.64	4.15	0.43	3.88	10.13	6.03	1.78	5.25	10.41	6.59	0.92	6.59	2.23	1.57	29.41	11.07	62.23	-	1.22
Protocatechuic acid	1.61	3.44	15.57	3.64	4.51	2.19	0.90	9.72	2.03	2.14	2.39	20.68	4.70	5.81	2.52	5.81	4.76	4.00	13.44	5.52	1.31	0.76	15.94
Sinapic acid	-	-	-	-	-	-	-	-	-	-	0.04	0.05	0.08	0.07	0.05	0.07	0.07	0.06	-	0.09	-	-	0.38
Syringic acid	1.70	1.12	1.10	0.79	2.43	1.40	0.97	2.29	2.32	1.20	0.30	-	-	1.59	0.55	1.59	1.71	0.53	1.35	0.70	-	0.35	0.11
Vanillic acid	0.51	1.40	1.99	1.32	1.64	0.45	0.23	8.55	0.64	0.51	0.14	1.02	1.19	1.22	0.16	1.22	2.07	0.35	0.58	0.24	0.12	0.25	0.53
**Phenolic aldehyde**																							
Vanillin	0.04	0.04	0.82	0.05	0.50	0.06	0.03	0.34	0.03	0.08	0.03	-	1.21	0.21	0.12	0.21	0.19	0.06	0.28	0.10	0.60	1.06	0.03
**Flavan-3-ols**																							
Epicatechin	-	-	-	-	-	-	-	-	0.18	-	-	-	-	-	-	-	-	-	-	-	-	-	-
**Flavanone**																							
Naringenin	-	-	-	-	-	-	-	-	-	-	-	-	-	0.97	12.03	0.97	0.25	-	-	-	-	-	0.17
**Flavones**																							
Chrysin	0.44	-	-	1.49	30.80	-	0.03	-	-	-	0.25	-	40.07	-	1.49	-	1.26	1.72	9.03	-	4.85	-	-
**Flavonols**																							
Quercetin	9.54	12.67	-	12.43	-	22.59	0.89	-	9.31	10.55	7.59	-	-	-	10.26	-	24.09	-	-	46.33	-	-	7.83
Kaempferol	1.99	11.03	-	7.25	-	4.64	-	-	4.68	3.64	1.15	-	-	19.41	48.60	19.41	4.11	-	-	4.17	-	-	15.43
**Stilbenoid**																							
Resveratrol	0.04	-	-	-	-	-	-	-	0.03	0.02	0.02	-	0.90	-	0.02	-	-	-	0.12	-	0.14	-	-

Table S6. Spectra bands intensities of vinegars.

Regions	RGBV [a]	RGBVH [b]	WGBV [c]	RGV [d]	WGV [e]	FV [f]
3100–3050	0.0121 ± 0.0032a	0.0160 ± 0.0023b	0.0102 ± 0.0016a	0.0027 ± 0.0005c	0.0022 ± 0.0005c	0.0016 ± 0.0013c
2940–2840	0.0252 ± 0.0061a	0.0324 ± 0.0047b	0.0221 ± 0.0030a	0.0087 ± 0.0012c	0.0075 ± 0.0012c	0.0065 ± 0.0022c
1730–1700	0.0325 ± 0.0009a	0.0229 ± 0.0066b	0.0305 ± 0.0017ab	0.0326 ± 0.0037a	0.0294 ± 0.0046ab	0.0225 ± 0.0108b
1425–1380	0.0375 ± 0.0074a	0.0433 ± 0.0044a	0.0329 ± 0.0041a	0.0187 ± 0.0021b	0.0163 ± 0.0025b	0.0127 ± 0.0050b
1300–1260	0.0470 ± 0.0062a	0.0469 ± 0.0045a	0.0432 ± 0.0039a	0.0312 ± 0.0034b	0.0279 ± 0.0045bc	0.0205 ± 0.0102c
1080–1040	0.0976 ± 0.0334a	0.1484 ± 0.0326b	0.0835 ± 0.0139a	0.0074 ± 0.0023c	0.0058 ± 0.0014c	0.0056 ± 0.0012c
825–810	0.0156 ± 0.0052a	0.0245 ± 0.0055b	0.0130 ± 0.0021a	0.0006 ± 0.0001c	0.0004 ± 0.0001c	0.0006 ± 0.0001c
790–770	0.0166 ± 0.0056a	0.0262 ± 0.0059b	0.0139 ± 0.0023a	0.0002 ± 0.0001c	0.0002 ± 0.0001c	0.0004 ± 0.0001c

[a] RGBV: red grape balsamic vinegars (n = 12), [b] RGBVH: red grape balsamic vinegars with honey (n = 5), [c] WGBV: white grape balsamic vinegars (n = 3), [d] RGV: red grape vinegars (n = 10), [e] WGV: white grape vinegars (n = 8), [f] FV: fruit vinegars (n = 5).

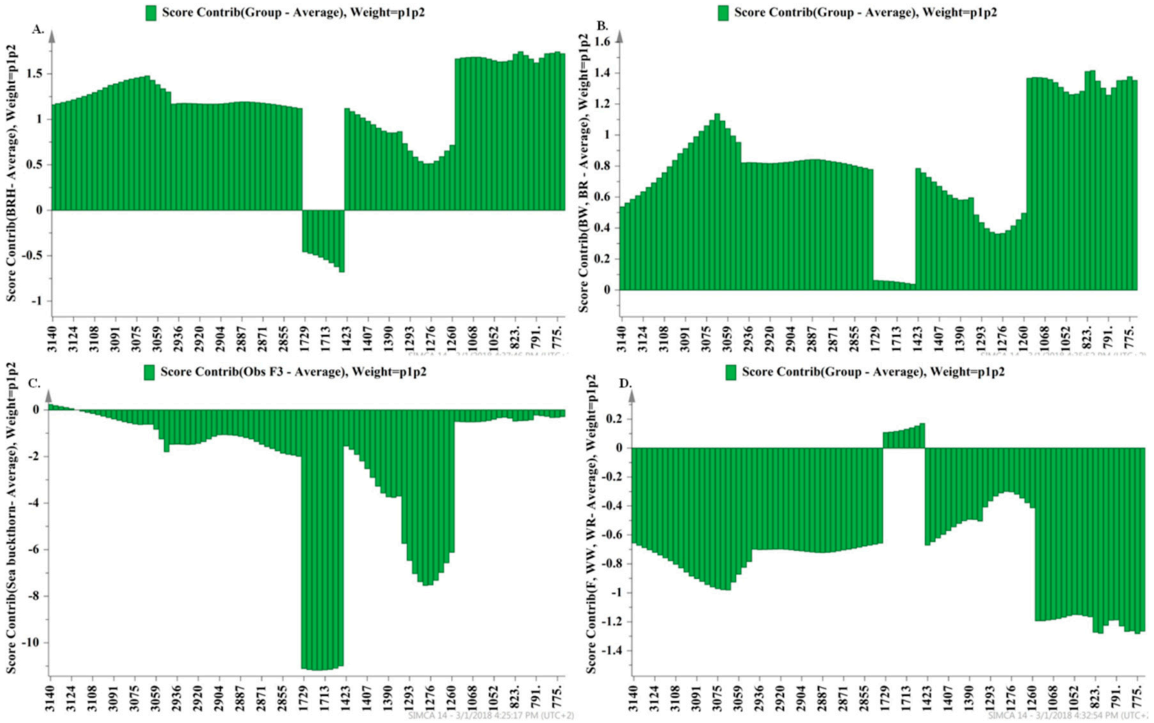

Figure S1. Contribution plots extracted from the PCA model. (**A**) Contribution plot of the balsamic vinegars with honey, (**B**) Contribution plot of the red and white balsamic vinegars, (**C**) Contribution plot of the vinegar with the embedded Sea Buckthorn, (**D**) Contribution plot of the common vinegars.

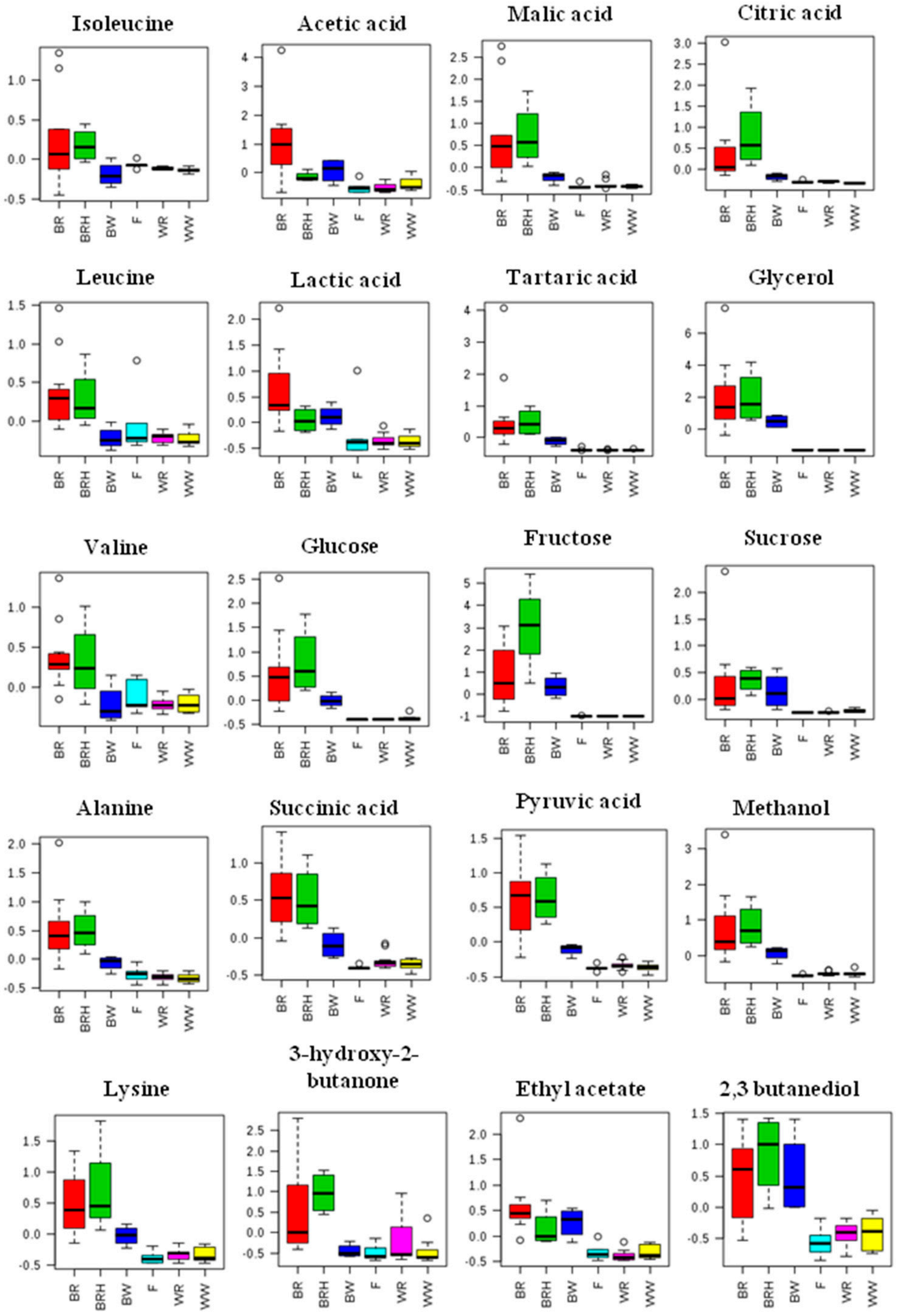

Figure S2. Box plots of the significant metabolites as pinpointed by Anova on the NMR data **(**BRH= balsamic vinegars with honey, BR= red balsamic vinegars, BW= white balsamic vinegars, WR= red common vinegars, WW= white common vinegars, F= common vinegars from fruits**)**
